# Bcr is a substrate for Transglutaminase 2 cross-linking activity

**DOI:** 10.1186/1471-2091-12-8

**Published:** 2011-02-10

**Authors:** Sun-Ju Yi, John Groffen, Nora Heisterkamp

**Affiliations:** 1 Section of Molecular Carcinogenesis, Division of Hematology/Oncology, Ms#54, Childrens Hospital Los Angeles, 4650 Sunset Boulevard, Los Angeles CA 90027, USA; 2 The Saban Research Institute of Childrens Hospital Los Angeles, CA 90027, USA; 3 Department of Pathology, Keck School of Medicine, University of Southern California, Los Angeles, CA 90033, USA

## Abstract

**Background:**

Breakpoint cluster region (Bcr) is a multi-domain protein that contains a C-terminal GTPase activating protein (GAP) domain for Rac. Transglutaminase 2 (TG2) regulates Bcr by direct binding to its GAP domain. Since TG2 has transglutaminase activity that has been implicated in the response to extreme stress, we investigated if Bcr can also act as a substrate for TG2.

**Results:**

We here report that activation of TG2 by calcium caused the formation of covalently cross-linked Bcr. Abr, a protein related to Bcr but lacking its N-terminal oligomerization domain, was not cross-linked by TG2 even though it forms a complex with it. A Bcr mutant missing the first 62 amino acid residues remained monomeric in the presence of activated TG2, showing that this specific domain is necessary for the cross-linking reaction. Calcium influx induced by a calcium ionophore in primary human endothelial cells caused cross-linking of endogenous Bcr, which was inhibited by the TG2 inhibitor cystamine. Treatment of cells with cobalt chloride, a hypoxia-mimetic that causes cellular stress, also generated high molecular weight Bcr complexes. Cross-linked Bcr protein appeared in the TritonX-100-insoluble cell fraction and further accumulated in cells treated with a proteasome inhibitor.

**Conclusions:**

Bcr thus represents both an interacting partner under non-stressed conditions and a target of transglutaminase activity for TG2 during extreme stress.

## Background

The breakpoint cluster region (Bcr) protein was originally identified as the amino-terminal part of a fusion protein including the Abl tyrosine kinase, which causes chronic myeloid leukemia and Ph-chromosome-positive acute lymphoblastic leukemia. The fusion of Bcr to Abl deregulates the tyrosine kinase activity of Abl [1]. Although the Bcr protein contributes a varying number of domains to the fusion protein, the N-terminal oligomerization domain of Bcr is considered to be the most critical component that allows the formation of homo-tetramer Bcr/Abl complexes and deregulates the Abl tyrosine kinase [2,3].

The normal (non-rearranged) *BCR *gene encodes a multidomain protein. Apart from the oligomerization domain, it additionally contains serine/threonine protein kinase, tandem DH-PH, C2 and GTPase activating protein (GAP) domains. The latter domain has a relatively well-described function: it down-regulates the activated GTP-bound conformation of the small G-protein Rac *in vitro *[4] and *in vivo *[5,6]. This function is shared by Abr, a related protein that also contains tandem DH-PH, C2 and GAP domains. However, Abr lacks the N-terminal oligomerization domain.

To understand how the GAP activity of Bcr is regulated, we performed a yeast two-hybrid screen with full-length Bcr and isolated transglutaminase 2 (TG2), an interesting multi-functional and multi-domain member of the transglutaminase family [7]. Unlike other transglutaminases, it is expressed in a variety of tissues and cells and also undergoes a GTP-binding/GTPase cycle. TG2 is located in the cytosol, in the nucleus, and on the surface of cells [8]. It appears to have multiple functions, including roles in differentiation, apoptosis, signal transduction, adhesion and migration, wound healing, inflammation and phagocytosis of apoptotic cells [8-11]. TG2 can adopt a closed conformation when its C-terminal domain is GTP-bound. We found that this conformation has decreased affinity for binding with Bcr. However, in the absence G-nucleotides, TG2 binds to Bcr and is able to inhibit the Bcr GAP activity towards Rac [7].

Transglutaminases exhibit several enzymatic activities. These include transamidation reactions (cross-linking, amine incorporation and acylation) as well as esterification, deamidation and isopeptidase activities [8,12-14]. TG2 is activated in the presence of high concentrations of calcium and converts to an open conformation. However, TG2 transglutaminase activity is latent under normal conditions because the steady-state concentration of calcium in the cytoplasm is low. It only becomes activated under pathogenic conditions and/or extreme cellular stress [15-18].

Because Bcr and TG2 can form a direct protein complex, we considered the possibility that Bcr could also act as a substrate for TG2. We here show that Bcr but not the related Abr protein is a substrate of TG2 *in vitro *and in cells under conditions of cellular stress. Interestingly, the presence of the N-terminal oligomerization domain in Bcr was responsible for this differential modification, which may lead to its degradation under conditions of extreme stress.

## Results

### TG2 cross-links Bcr

TG2 can adopt alternate conformations depending on the presence of G-nucleotides or calcium. We previously showed that TG2 interacts with Bcr when TG2 is in the open, non-G-nucleotide bound conformation, which is also the conformation adopted in the presence of calcium [7]. Therefore, we investigated whether Ca^2+ ^could affect the interaction between TG2 and Bcr.

**Figure 1:**
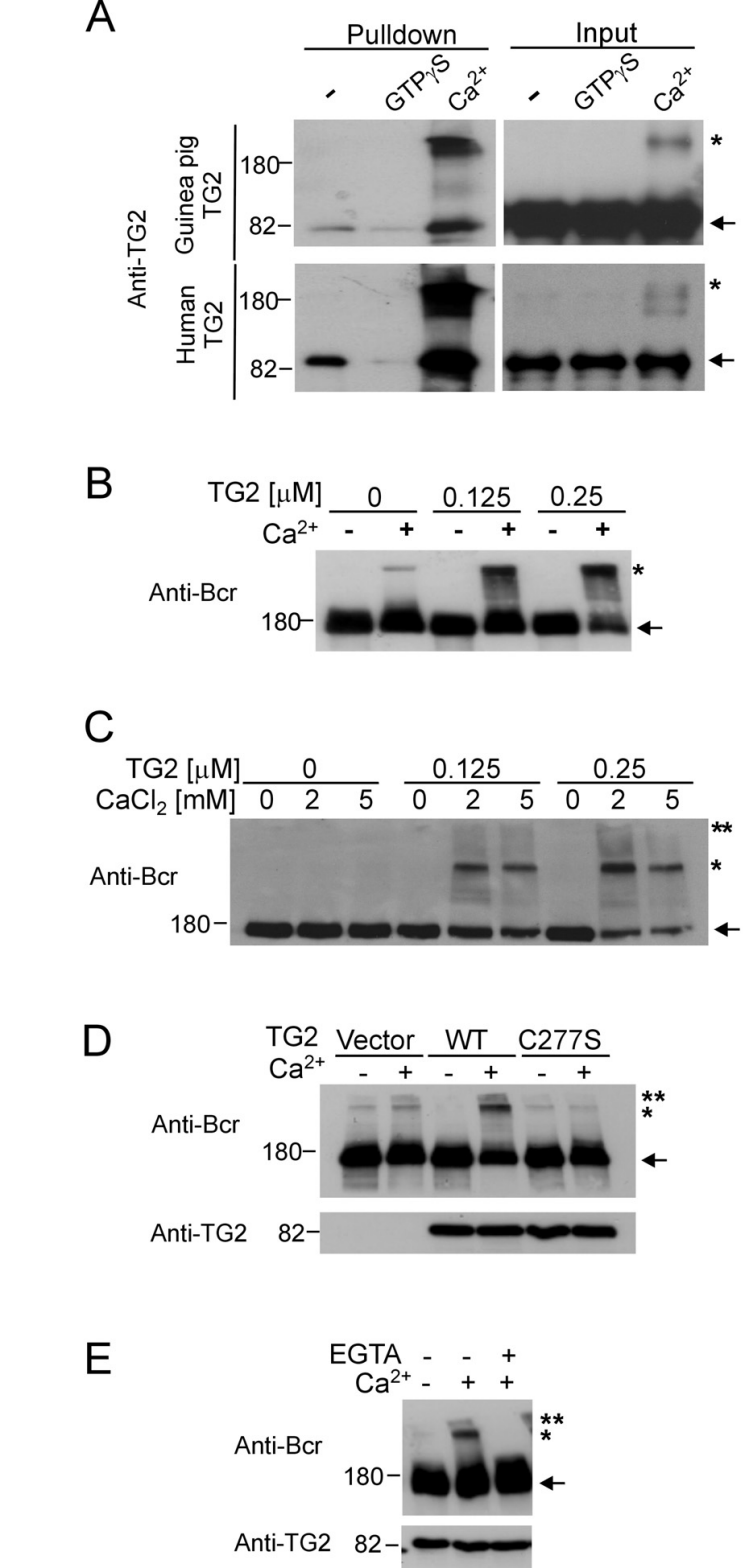
**TG2 cross-links Bcr**. **A**) GST-BcrGAP was incubated with guinea pig liver TG2 (Guinea pig TG2) or His_6_-tagged human TG2 (Human TG2) that was loaded with GTPγS, or exposed to Ca^2+ ^or treated with nothing. GST-BcrGAP was pulled down with glutathione-agarose beads and TG2 bound to it detected by Western blot analysis. **B**) Bcr-transfected COS-1 cell lysates incubated with the indicated amounts of guinea pig TG2 with or without 5 mM CaCl_2_. **C**) HeLa cell lysates treated with CaCl_2 _and guinea pig TG2 as indicated. **D**) COS-1 were transfected with Bcr and the indicated TG2 constructs. Cell lysates were treated with or without CaCl_2_. **E**) Bcr and TG2-transfected cell lysates were incubated with EGTA and/or CaCl_2 _as indicated for 2 h at 37°C. Antibodies used for Western blotting are indicated to the left. Arrows indicate guinea pig TG2 or His_6_-tagged human TG2 (A) or Bcr monomer (B-E); single asterisks indicate the top of separating gel; double asterisks indicate the wells of the stacking gel.

As shown in Figure 1A, we pre-incubated TG2 with Ca^2+ ^or with GTPγS, then added GST-BcrGAP, and performed a GST pull-down assay to investigate the binding of Bcr and TG2 by immunoblotting with antibodies to TG2. GTPγS loading of both guinea pig TG2 as well as recombinant His-tagged TG2 reduced its binding to BcrGAP (Figure 1A), in agreement with our previous results [7].

The presence of calcium is known to activate TG2 and cause auto-crosslinking [19]. Interestingly, in the presence of Ca^2+ ^there was increased binding of the TG2 monomer to BcrGAP and, moreover, the TG2 multimer also was pulled down with BcrGAP (Figure 1A).

Since the transamidase activity of TG2 is Ca^2+^-dependent, we next assessed whether Bcr is a substrate of TG2. We transfected COS-1 cells with Bcr, added guinea pig liver TG2 to cell lysates, and performed a transglutaminase assay in the presence or absence of Ca^2+^. As shown in Figure 1B, addition of exogenous TG2 produced high molecular weight Bcr aggregates in a dose- and calcium-dependent manner. Also, we observed high molecular weight aggregates of endogenous Bcr in HeLa cell lysates in the presence of guinea pig TG2 (Figure 1C). To investigate if this cross-linked Bcr results from the transglutaminase activity of TG2, TG2 wild type or C277S (a transamidase-defective mutant) and Bcr were co-transfected into COS-1 cells, after which a transamidase assay was performed. As shown in Figure 1D, the TG2 C277S mutant did not induce aggregate formation of Bcr, whereas expression of the wild type TG2 increased the levels of cross-linked Bcr. To confirm that the cross-linking activity is Ca^2+^-dependent, cell lysates were also pretreated with EGTA, a chelator of Ca^2+^. Figure 1E shows that the cross-linking reaction was blocked by 5 mM EGTA. These results indicate that Bcr aggregate formation requires transglutaminase activity of TG2 and Ca^2+^. Since Bcr naturally forms homo-tetramers, these high molecular weight complexes will consist of Bcr monomers but could also contain TG2, since it was recovered in a pull-down reaction with the Bcr GAP domain (Figure 1A), as well as other unidentified proteins.

### Bcr oligomerization domain is required for cross-linking by TG2

**Figure 2:**
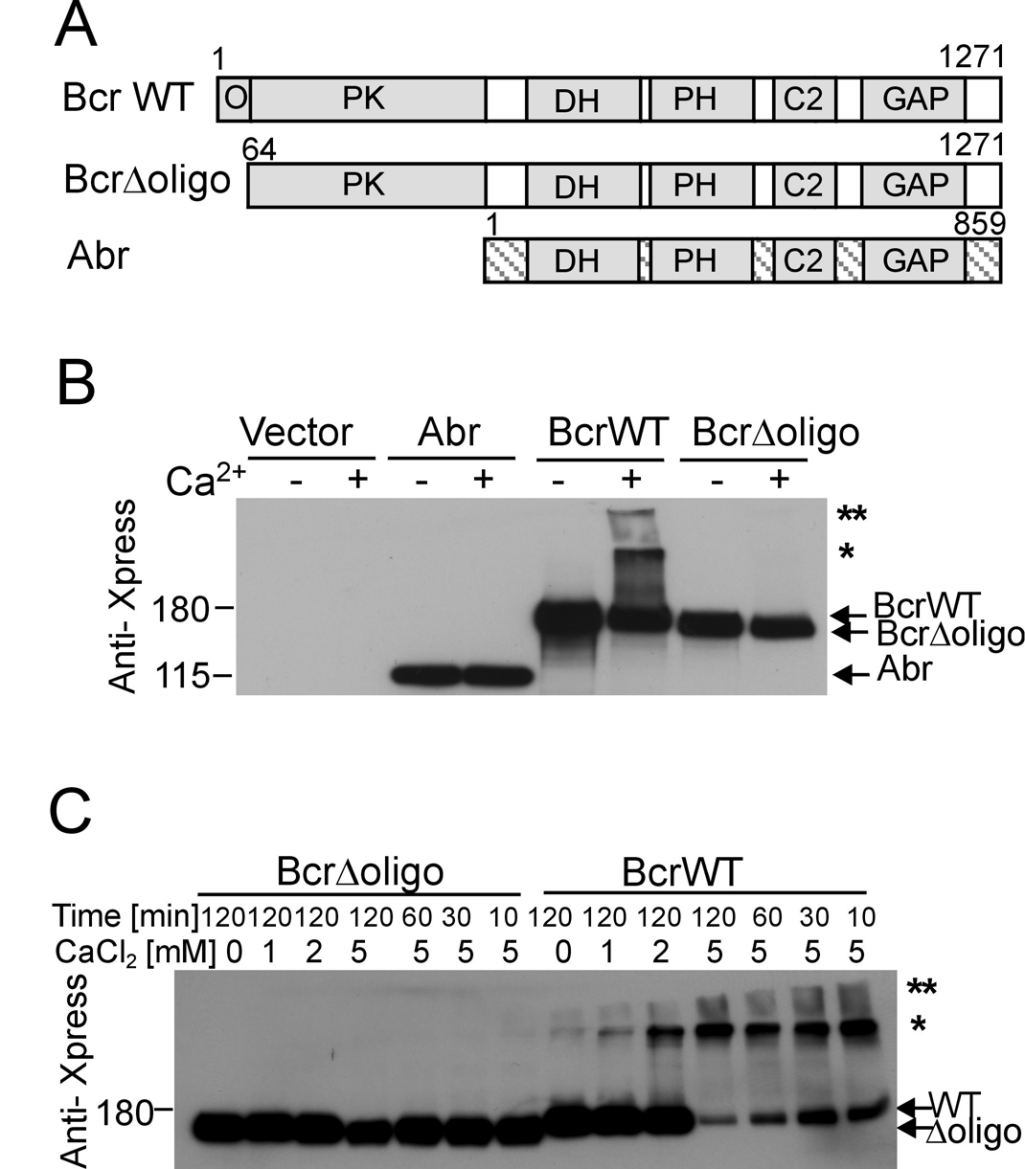
**Oligomerization domain of Bcr mediates cross-linking by TG2**. **A**) Schematic view of Bcr and Abr constructs. O, oligomerization domain; PK, protein serine/threonine kinase domain; DH, Dbl homology domain; PH, Pleckstrin homology domain; C2, domain with homology to C2 domains that are calcium-dependent, protein or phospholipid binding; GAP, GTPase activating protein. **B**) Xpress-tagged Abr, Bcr wild type (WT), or Bcr mutant lacking the oligomerization domain (Δoligo) were co-transfected with TG2. Lysates were incubated with or without 5 mM CaCl_2_. **C**) Lysates of cells co-transfected with TG2 and Xpress-tagged BcrWT or BcrΔoligo were incubated with CaCl_2 _for the indicated times. Asterisks, as in Figure 1.

Bcr is a large protein containing several domains (Figure 2A). Abr (Active Bcr Related) also functions as a GAP for Rac and is the only protein that shares a high degree of homology with Bcr [20]. The main difference between the two proteins is, that Abr lacks a domain that has homology to the N-terminal end of Bcr. This region in Bcr includes a serine/threonine kinase domain of unknown significance and a coiled-coil domain that allows Bcr to form tetramers. We previously showed that the Abr GAP domain can bind to TG2 [7]. However, as shown in Figure 2B, Abr was not detectably cross-linked by TG2.

We therefore asked if the N-terminal coiled-coil domain of Bcr could be responsible for allowing TG2 to cross-link Bcr. A construct that lacked the 62 N-terminal amino acids was co-expressed with TG2 and compared to the wild type Bcr protein. As shown in Figure 2B, whereas Xpress-tagged Bcr wild type was cross-linked, the protein lacking the coiled-coil region clearly was not. We confirmed this result by increasing the reaction time or concentration of Ca^2+^: no cross-linking of the BcrΔoligo protein was detected at any length of incubation or concentration of calcium. In contrast, increased concentrations of calcium and prolonged reaction times resulted in progressively increased Bcr appearing in high molecular weight complexes. After 2 hours with 5 mM CaCl_2_, very little monomeric Bcr remained (Figure 2C). These results indicate that the oligomerization domain of Bcr is cross-linked by TG2.

### Bcr is cross-linked in cells under extreme stress

**Figure 3:**
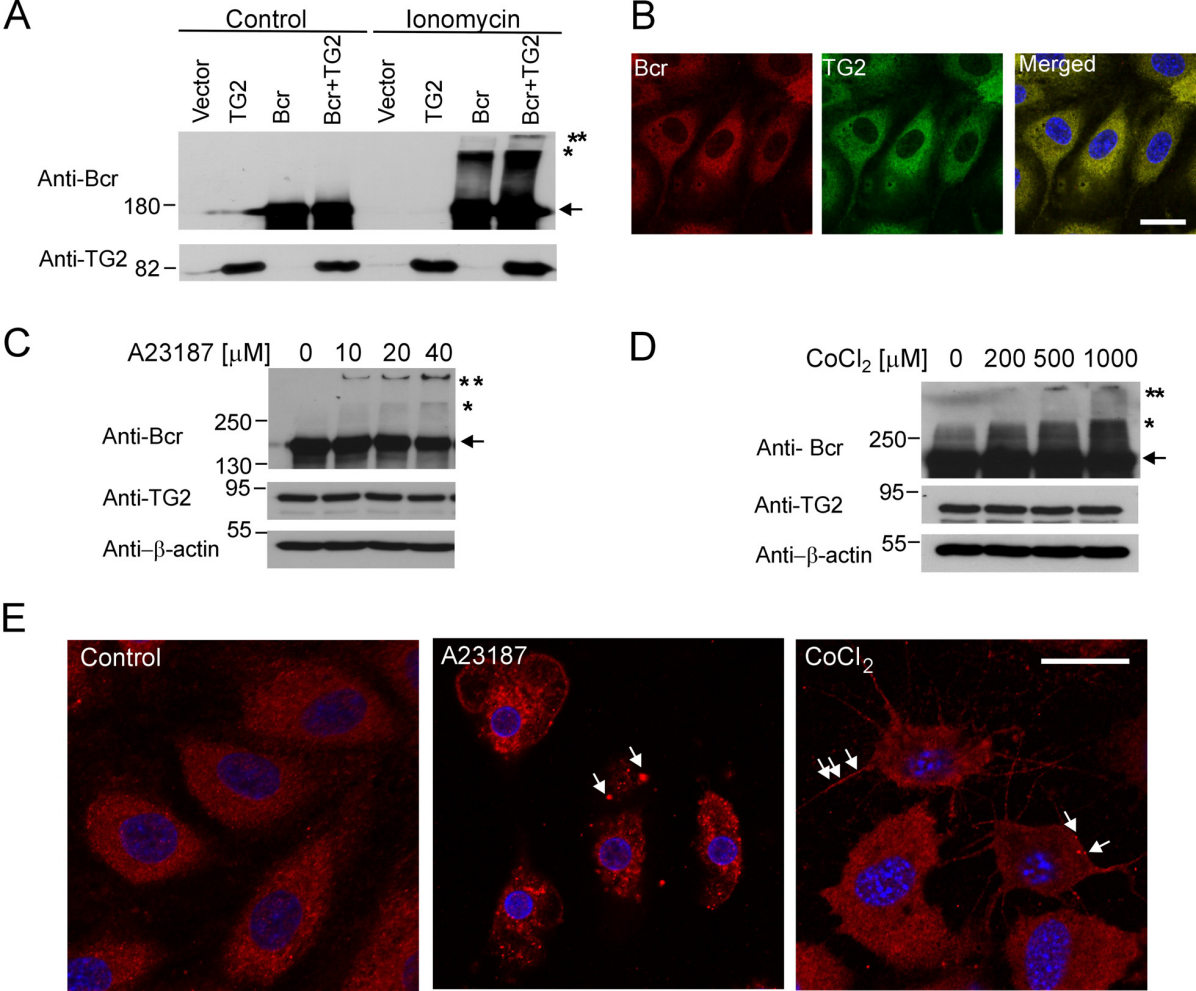
**Ca**^**2+ **^**influx or CoCl**_**2 **_**stimulates Bcr aggregation in cells**. **A**) COS-1 cells transfected with the indicated plasmids were treated with DMSO (Control) or 1 μM ionomycin for 1 h. Antibodies used for Western blots are indicated to the left. **B**) Localization of Bcr and TG2 in HPAECs using confocal microscopy. Bar, 20 μm. **C, D**) HPAECs were incubated with the indicated concentrations of A23187 for 1 h (C) or CoCl_2 _for 24 h (D). Antibodies used for Western blotting are indicated to the left. **E**) Cellular localization of Bcr in HPAECs after treatment with DMSO (Control), 20 μM A23187 for 1 h or 1000 μM CoCl_2 _for 24 h using confocal microscopy. DAPI was used to stain nuclei. Arrows point to some of the intracellular Bcr aggregates. Bar, 20 μm.

In the experiments described above, calcium was added to protein lysates. The transglutaminase activity of TG2 can also be activated in the presence of increased levels of intracellular Ca^2+^. To examine TG2-induced aggregation of Bcr in cells, COS-1 cells were transfected with Bcr alone or together with TG2 and treated with ionomycin, a calcium ionophore, which induces cell death at high concentrations [21]. As shown in Figure 3A, co-expression of TG2 with Bcr caused a significant increase of high molecular weight Bcr complexes in cells. Ionomycin treatment also caused Bcr cross-linking in cells transfected only with Bcr, likely through activation of endogenous TG2 (Figure 3A, lane Bcr/ionomycin).

To examine cross-linking of endogenous Bcr, we selected primary human pulmonary artery endothelial cells (HPAECs), since this cell type expresses substantial amounts of TG2 [7,22]. In addition, TG2 plays a role in endothelial cell adhesion, proliferation and apoptosis [23]. As shown in Figure 3B, these cells clearly expressed Bcr protein, and Bcr co-localized with endogenous TG2 in the cytosol. Exposure of HPAEC to A23187, a different calcium ionophore, induced Bcr cross-linking, as was evident by the appearance of high molecular weight Bcr complexes, and this was accompanied by a reduction in the amount of monomers (Figure 3C). These complexes were detected as a smear of Bcr-immunoreactive material at the top of the separation gel and even in the wells of the stacking gel, in an A23187 dose-dependent manner.

CoCl_2 _is a hypoxia-mimetic agent, because like hypoxia, it can decrease the degradation and cause subsequent accumulation of hypoxia-inducible factor (HIF)-1α, a critical regulator of the cellular response to hypoxia [24]. Also, CoCl_2 _induces cell death [24-28]. It was also recently reported that hypoxia induces the expression of TG2 via HIF-1α in some cancer cell types, but not in others [29]. To investigate the effect of this type of extreme stress on Bcr cross-linking by TG2, we exposed HPAECs to increasing concentrations of this heavy metal. As shown in Figure 3D, the treatment with high concentrations of CoCl_2 _caused the appearance high molecular weight Bcr aggregates, suggesting that hypoxic stress can also cause covalent adducts on Bcr. CoCl_2 _treatment had no effect on the level of TG2 expression in HPAECs (Figure 3D, anti-TG2 Western blot).

Treatment with A23187 or CoCl_2 _visibly stressed the cells, which showed signs of apoptosis as determined by FACS (not shown). Consistent with this, DAPI staining showed nuclear condensation and fragmentation (Figure 3E). Interestingly, endogenous Bcr showed a prominent punctuate staining, especially in A23187 treated cells (Figure 3E, arrows). This is consistent with CoCl_2 _or A23187 causing accumulation of Bcr aggregates in HPAECs that are undergoing cell death.

### Cross-linked Bcr is removed by proteasomal degradation

**Figure 4:**
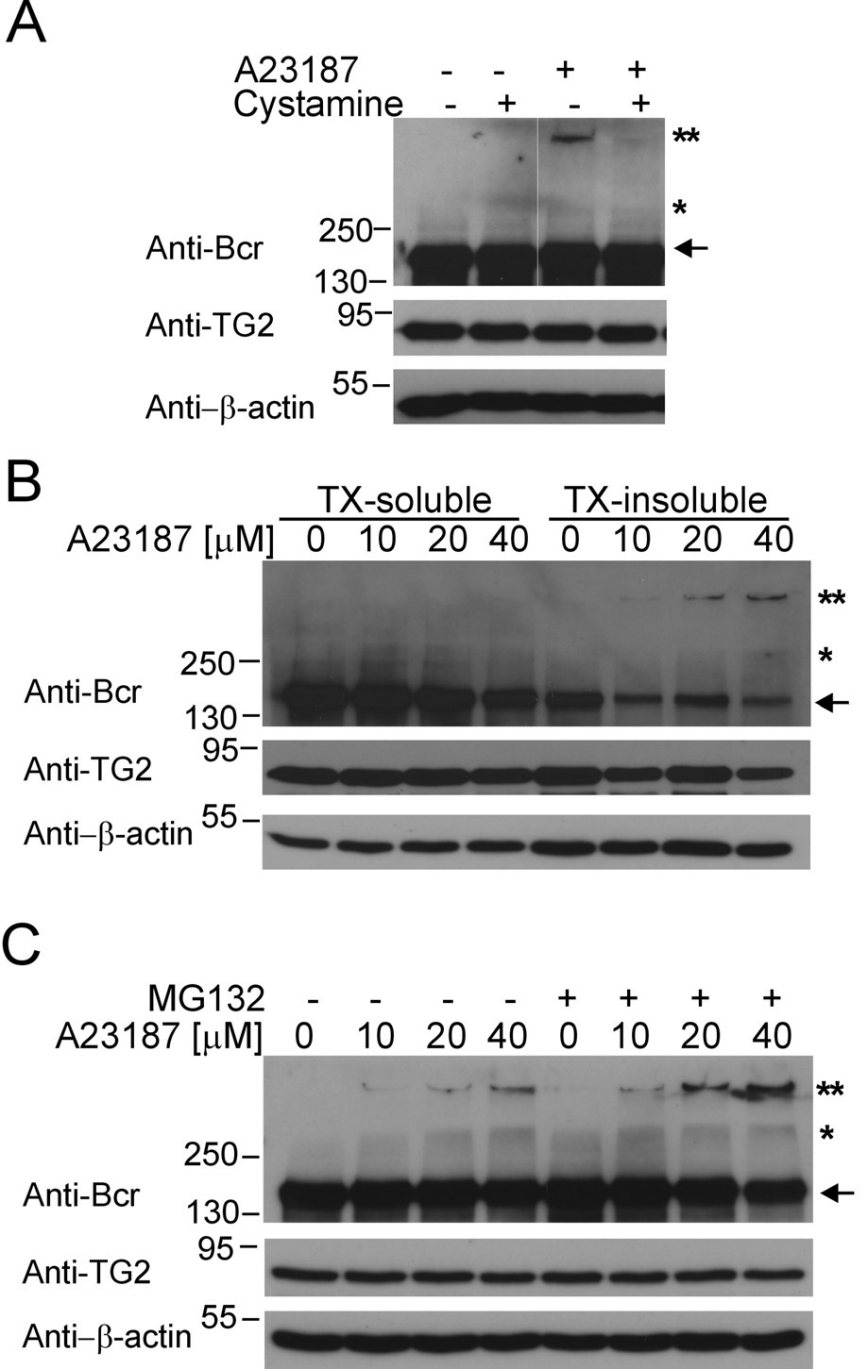
**Bcr aggregates are degraded by a proteasome-dependent pathway**. **A**) HPAECs were pretreated with 100 μM cystamine for 30 min, then incubated with 20 μM A23187 for 1 h. Cell lysates were subjected to Western blot analysis with the antibodies indicated to the left. **B**) After incubation with A23187 for 1 h, HPAECs were lysed and separated into Triton X-100 soluble (TX-soluble) or insoluble (TX-insoluble) fractions. The same amount of protein was subjected to Western blot for Bcr, TG2, or β-actin. **C**) HPAECs were pretreated with a proteasome inhibitor, MG132 (50 μM for 30 min), followed by incubation with the indicated concentrations of A23187. Antibodies used for Western blotting of lysates are indicated to the left. Asterisks, as in Figure 1.

As shown in Figure 4A, pretreatment with a competitive TG2 inhibitor, cystamine, dramatically suppressed the appearance of Bcr high molecular weight aggregates, further confirming that the formation of these complexes is dependent on TG2 activity. Since the solubility of some proteins changes after modification by TG2, including monomeric forms and aggregates [30,31], we separated cell lysates into TritonX-100 soluble and insoluble fractions. As shown in Figure 4B, the high molecular weight Bcr aggregates were mainly present in the TritonX-100-insoluble fraction, and more Bcr monomers were present in the TritonX-100-soluble fraction.

It has been shown that TG2 promotes the proteasomal degradation of some proteins including nucleophosmin and PTEN [31,32]. To investigate if the high molecular weight Bcr aggregates are degraded in a proteasome-dependent pathway, HPAECs were pretreated with MG132, a proteasomal inhibitor, before exposure to the calcium ionophore. Interestingly, pretreatment with MG132 caused accumulation of the high molecular weight aggregates of Bcr (Figure 4C). These results suggest that Bcr aggregates, resulting from cross-linking by TG2, are ultimately disposed of by proteasomal degradation.

## Discussion

Binding of guanine nucleotides or of Ca^2+ ^to TG2 is known to inversely regulate its conformation and its transglutaminase activity: whereas Ca^2+^-bound TG2 has an open conformation and is active as a transglutaminase, TG2 bound to G-nucleotides is enzymatically inactive, with a closed conformation [8,16,33]. Although Bcr binds preferentially to the open conformation of TG2, the interaction is not dependent upon TG2 being active as a transglutaminase because the interaction also occurred with the catalytically inactive TG2 C277S mutant [7]. Our current results demonstrate that Bcr also binds quite strongly to the activated, calcium-bound form of TG2. Together, these results suggest that under normal physiological conditions, TG2 interacts with Bcr even though the calcium concentration is not sufficient for activation of the transglutaminase activity. Under conditions of cellular stress caused by high calcium influx, the calcium-bound, enzymatically active form of TG2 then uses Bcr as a substrate for cross-linking.

Apart from high calcium concentrations, other cellular stressors that have been reported to involve TG2 activation include exposure to bacterial endotoxin and glutamate-mediated excitotoxic damage of neuronal cells [34]. However, we did not detect Bcr aggregates in primary rat neurons exposed to glutamate or bone marrow-derived macrophages treated with LPS (not shown). Also, Bcr aggregates were only detected in HPAECs stressed by CoCl_2 _or A23187 treatment and not by other stimulants including LPS, TNF-α, and thrombin (data not shown). Therefore the formation of Bcr aggregates may be specific only for certain types of cell stress that lead to cell death.

To the best of our knowledge, the biological effect of TG2 cross-linking due to calcium overload has not been conclusively defined for any protein. However, based on what is known about Bcr structure and function, we can envision three possible consequences of Bcr cross-linking under conditions of calcium influx and apoptosis. One is a role that was postulated in general for the consequences of TG2 cross-linking activity under conditions of cell death: TG2 activity could produce a protein-rich "shell" that would prevent leakage of cellular components to the extracellular space and, through this, reduce inflammation that would otherwise be provoked by cell debris [35-37]. Bcr contains a C2 domain that, in other proteins, is a calcium-binding module. The adjacent PH domain was recently shown to be able to bind to keratin 10, β-tubulin and collagen IV [38]. Because the high molecular weight Bcr aggregates found in our study were mainly present in the TritonX-100-insoluble fraction, one could speculate that Bcr, due to multiple cellular interactions including its own capacity to form homo-tetramers, acts as a calcium-induced scaffold for the formation of high molecular weight aggregates including elements of the cytoskeleton. This function would be consistent with the overall activity of Bcr to negatively regulate inflammation *in vivo *[6,7].

A second speculative role is in the area of vesicle transport. The PH domain of Bcr was shown to bind to specific mono-phospholipids PtdIns(3)P and PtdIns(4)P that are present in early endosomal membranes and in the Golgi, respectively [38]. Moreover, Bcr binds to the endosomal sorting protein TSG101 [39], which is also present in exosomes [40]. Since cross-linking of Bcr could affect its GAP activity on Rac, this in turn could potentially affect the trafficking of extracellular TG2. Finally, our results suggest that cross-linked Bcr is targeted for degradation. Since Bcr is a negative regulator of Rac, its removal would lead to increased levels of activated Rac, which has been associated with both decreased and increased apoptosis, depending on the cell type.

Bcr contains a unique N-terminal coiled-coil domain that has been a focus of significant interest because it is responsible for deregulation of the tyrosine kinase activity of Abl within the Bcr/Abl fusion protein that causes the development of some types of leukemia. Interestingly, this domain also allows the formation of Bcr homodimers and tetramers [2,3,41,42]. We here found that it plays an important role in the formation of Bcr aggregates. The domain encompasses residues 2-63, which includes two lysine and five glutamine residues that could be involved in the cross-linking reaction, but the exact requirements for cross-linking remain to be determined. It will also be of interest to address if TG2 is able to cross-link the N-terminal oligomerization domain of the Bcr/Abl oncoprotein.

## Conclusions

We recently showed that TG2 directly interacts with Bcr and regulates the GAP activity of Bcr under normal physiological conditions. Here, we present data showing that Bcr is a substrate for TG2 transglutaminase activity *in vitro *and in cells. Under extreme stress, TG2 cross-links Bcr into high molecular weight complexes which are removed by a proteasomal degradation pathway. These results suggest that Bcr is regulated by TG2 via different mechanisms, depending on the physiological conditions in the cell.

## Methods

### Plasmids and Antibodies

Full length human TG2 wild type, TG2 C277S cDNAs and Xpress-tagged Bcr wild type have been described previously [7]. To generate Xpress-tagged BcrΔoligo, we isolated the N-terminal end of Bcr with deletion of amino acid residues 2-63 from a previously described 474 bp subclone by digestion with Eag I × Xho I [43]. The 3.5 kb 3' end of *BCR *was isolated from a cDNA clone in pSK (B1/SK) by digestion with Xho I × Xba I. These two fragments were combined in pcDNA3.1/HisB digested with Not I × Xba I. Xpress-tagged Abr was constructed in a 3-way ligation between a 5' 0.45 kb BamHI-BstEII, a 3' 2.2 kb BstEII-EcoRI fragment and vector pcDNA/HisC digested with BamHI × EcoRI. The amino-terminal Abr end is the one expressed on the non-neuronal isoform. Bcr (N-20), Xpress and TG2 antibodies were from Santa Cruz Biotechnology (Santa Cruz, CA), Invitrogen (Carlsbad, CA) and Lab Vision (Fremont, CA), respectively. MG132 and monoclonal Bcr (Ab-2) antibodies were from EMD Chemicals (Gibbstown, NJ).

### Cell culture

COS-1 and HeLa cells were obtained from the American Type Culture Collection (Manassas, VA). Human pulmonary artery endothelial cells (HPAECs) were from Invitrogen, and were cultured in Medium 200 with low serum growth supplement according to the manufacturer's protocol. They were used at passage 4-6.

### GST pull-down assay

Recombinant GST-BcrGAP and His_6_-tagged human TG2 were purified from *E. coli *as described [4,44]. For GTPγS or Ca^2+ ^loading of TG2 and *in vitro *binding assays, 12.5 pmol of guinea pig TG2 (Sigma, St. Louise, MO) or His_6_-tagged TG2 was incubated in 25 mM Tris, pH 7.4, 1 mM EGTA, 1 mM EDTA, 1 mM DTT, 2 mM MgCl_2_, 100 mM NaCl, 0.1% Igepal, 1 mM phenylmethylsulfonyl fluoride (PMSF), 10 μg/ml aprotinin, 10 μg/ml leupeptin, 10% glycerol, and 100 μM GTPγS or 5 mM CaCl_2 _for 20 min at 30°C. 12.5 pmol of GST-BcrGAP was added and allowed to form a complex for 1 h at 4°C, followed by incubation with glutathione-agarose beads for 1 h at 4°C.

### Transfection and cross-linking assay

COS-1 cells were transfected with Plus reagent and Lipofectamine (Invitrogen) according to the manufacturer's instructions and grown for 2 days prior to the assay. For cross-linking assays, cells were lysed in transglutaminase assay buffer (50 mM Tris, pH 8.0, 150 mM NaCl, 1% Triton X-100, 1 mM DTT, 1 mM PMSF, 10 μg/ml aprotinin, 10 μg/ml leupeptin, 1 μg/ml pepstatin). Cell lysates (50 μg) were incubated with or without 5 mM CaCl_2 _for 2 h at 37°C, based on previously reported concentrations of 5 mM or more of CaCl_2 _added to *in vitro *assays, using cell extracts or purified proteins [29,45]. In some experiments, guinea pig TG2 or EGTA was added to the reactions.

### Treatment with calcium ionophores or CoCl_2_

Transfected COS-1 cells were treated with 1 μM ionomycin for 1 h. HPAECs were treated with 10-40 μM A23187 for 1 h or with CoCl_2 _for 24 h and lysed with SDS lysis buffer (50 mM Tris, pH 6.8, 2% SDS, 10% glycerol). For some experiments, cells were pretreated with cystamine or MG132 for 30 min. For separation of TritonX-100-soluble and insoluble fractions, cells were lysed in 50 mM Tris, pH 7.6, 150 mM NaCl, 1% Triton X-100, 5 mM EDTA, 1 mM PMSF, 10 μg/ml aprotinin, 10 μg/ml leupeptin, 1 μg/ml pepstatin and centrifuged at 16000 g for 15 min. The supernatants were collected as TritonX-100-soluble fractions and insoluble pellets were solubilized in SDS lysis buffer as TritonX-100 insoluble fractions.

### Immunofluorescence

HPAECs were grown on fibronectin-coated coverslips (Fisher Scientific) for 1-2 days and then treated with A23187 or CoCl_2. _Cells were washed twice with PBS and fixed in 4% paraformaldehyde (Electronic Scientific Co., Hatfield, PA) (15 min at room temperature [RT]), followed by permeabilization in 0.2% Triton X-100 (15 min, RT). Cells were blocked in 1% bovine serum albumin in PBS and stained with TG2 antibodies (Lab vision Ab-4, 5 μg/ml) and Bcr antibodies (EMD chemicals Ab-2, 1 μg/ml) in blocking solution overnight at 4°C, followed by incubation with Cy3-conjugated anti-mouse IgG and FITC-conjugated anti-rabbit IgG antibodies (Jackson ImmunoResearch Laboratories, Inc, West Grove, PA). After mounting in Vectashield containing 4',6'-diamidino-2-phenylindole (DAPI; Vector Laboratories, Burlingame, CA), cell images were acquired with a Zeiss 710 confocal microscope.

## Abbreviations

Bcr: breakpoint cluster region; TG2: transglutaminase 2; GAP: GTPase activating protein; HPAECs: human pulmonary artery endothelial cells; PMSF: phenylmethylsulfonyl fluoride; RT: room temperature; DAPI: 4',6'-diamidino-2-phenylindole; DMSO: dimethyl sulfoxide.

## Authors' contributions

SY participated in the study design, performed all experiments described here and wrote a draft of the manuscript. JG contributed ideas for experiments; NH participated in study design, provided ideas for experiments and wrote the manuscript. All authors read and approved the final manuscript.
